# Factors associated with quality of life in retirement: a systematic review

**DOI:** 10.47626/1679-4435-2022-876

**Published:** 2023-02-13

**Authors:** Isadora Gabriella Paschoalotto Silva, Veronica Francisqueti Marquete, Iven Giovanna Trindade Lino, Vanessa Carla Batista, Gabriela Magnabosco, Maria do Carmo Fernandez Lourenço Haddad, Sonia Silva Marcon

**Affiliations:** 1 Programa de Pós-Graduação em Enfermagem, Universidade Estadual de Maringá, Maringá, PR, Brazil

**Keywords:** retirement, aging, health behavior, indicators of quality of life, systematic review, aposentadoria, envelhecimento, comportamentos relacionados com a saúde, indicadores de qualidade de vida, revisão sistemática

## Abstract

The purpose of this review was to determine the effects of retirement on quality of life and associated factors among older adults. This integrative review addressed the following question: what factors are associated with the health and quality of life of retired older adults? Biblioteca Virtual em Saúde and PubMed databases were searched using the following terms: retirement, quality of life, and health. Searches were conducted between June and December 2020. A total of 22 studies were included in the sample, categorized as follows: financial situation, social life, health conditions, and retirement preparation programs. The results indicate that quality of life among retirees is influenced by socioeconomic conditions, and the factors associated with this phenomenon differ according to culture, education, income, and professional category.

## Abstract (pt)

Conhecer os efeitos da aposentadoria na qualidade de vida de idosos aposentados e os fatores associados a esse fenômeno. Trata-se de revisão integrativa, com o seguinte questionamento: quais são os fatores associados à saúde e qualidade de vida do idoso aposentado? A busca foi realizada nas bases de dados Biblioteca Virtual em Saúde e Biblioteca Nacional de Medicina dos Estados Unidos utilizando os seguintes descritores: aposentadoria, qualidade de vida e saúde. As buscas foram realizadas no período de junho a dezembro de 2020. A amostra foi composta por 21 estudos, verificando-se quatro categorias: condições financeiras, convívio social, condições de saúde e programas de preparação para a aposentadoria. Os aposentados têm a qualidade de vida influenciada pelas condições socioeconômicas, sendo os fatores associados a esse fenômeno diferentes conforme a cultura, o ensino, a renda e as categorias profissionais.

## INTRODUCTION

Recent advances in technology and science have led to significant growth of the older adult population, both in Brazil and worldwide.^1^ Due to decreased mortality, increased life expectancy, and age structure changes,^2^ 18.5% of the total population of Latin America is projected to be older adults by 2050.^3^ Thus, it is concerning that, despite the demographic change, few policies and actions have been aimed at the needs of older adults.^4^

Although population aging is a worldwide phenomenon, it has a greater social and economic impact in developing countries than developed countries.^5^ For example, in developing countries, many older people must continue working to obtain income, which is often related to individual and/or family support. For people over 65 years of age, this can lead to health problems, depending on the work conditions, educational standards, education level, and job opportunities.^5,6^ Furthermore, it must be considered that the changes involved in the aging process are inevitable and occur naturally through physiological and biological factors, although they can also be pathological when disease is involved.^7^

Due to the significance attached to work in modern society - a means of achieving recognition and economic and social status^8^ - the retirement process can influence emotional and social dimensions, further impairing the functionality of retired people. It can lead to biophysiological, psycho-emotional, and socioeconomic vulnerability, in addition to unexpected behavior, psychopathologies, and new and inadequate attitudes.^4,9^

The increased social seclusion of retirement can restrict physical activity, impairing mobility, which accelerates the aging process and increases the incidence of chronic disease.^10^ All such changes make retirees less adaptable.^11^ According to one study, people who enter retirement without proper preparation cannot deal with the “excess” free time, which leads to feelings of uselessness.^11^

Faced with the upheaval that retirement can cause and its impact on health and quality of life, the objective of this study was to determine the effects of retirement on quality of life and associated factors among older adults.

## METHODS

This integrative review consisted of 6 stages: 1) determining the hypothesis/research question; 2) determining the inclusion and exclusion criteria and data collection methods; 3) categorizing the studies; 4) evaluating the selected studies; 5) interpreting the results; and 6) synthesizing the results.^12^

The research question was developed using the PICO strategy: Population (older adults), Interest (quality of life and health), and COntext (retirement). Thus, the question that guided the present study was “What factors are associated with the health and quality of life of retired older adults?” To answer the question, the following national and international electronic databases were searched for publications: Latin American and Caribbean Health Sciences Literature (LILACS), Biblioteca Virtual em Saúde, and the U.S. National Library of Medicine (PubMed). To obtain more recent findings, only full-text articles published between 2015 and 2020 were eligible.

A combination of the following indexed terms was used as a search strategy: “retirement”/“*aposentadoria*”, “quality of life”/“*qualidade de vida*” AND “health”/“*saúde*”. Articles published in English or Portuguese were eligible for inclusion. Database searches were conducted between June and December 2020.

**Figure 1:**
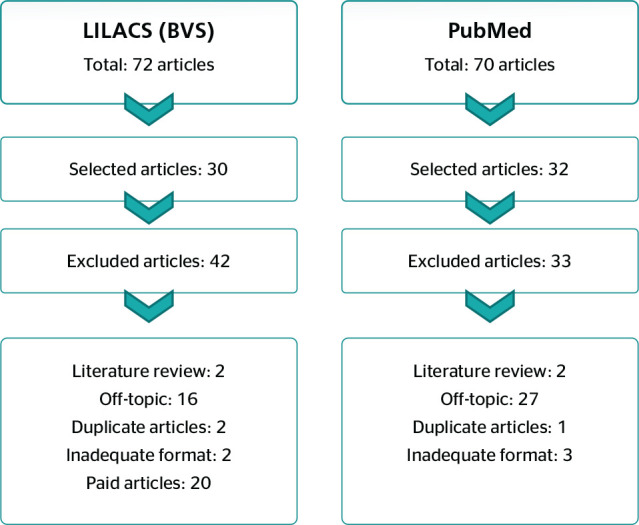
Search strategy and data collection. PRISMA (Preferred Reporting Items for Systematic Reviews and Meta-Analyses), Maringá, PR, Brazil, 2020. BVS = Biblioteca Virtual em Saúde; LILACS = Literatura Latino-Americana e do Caribe em Ciências da Saúde; PubMed = United States National Library of Medicine.

As further inclusion criteria, only open-access articles on retirement with the full text available were eligible. Review articles, experience reports, dissertations, theses, letters to the editor, and editorials were excluded (Figure 1).

**Figure 2:**
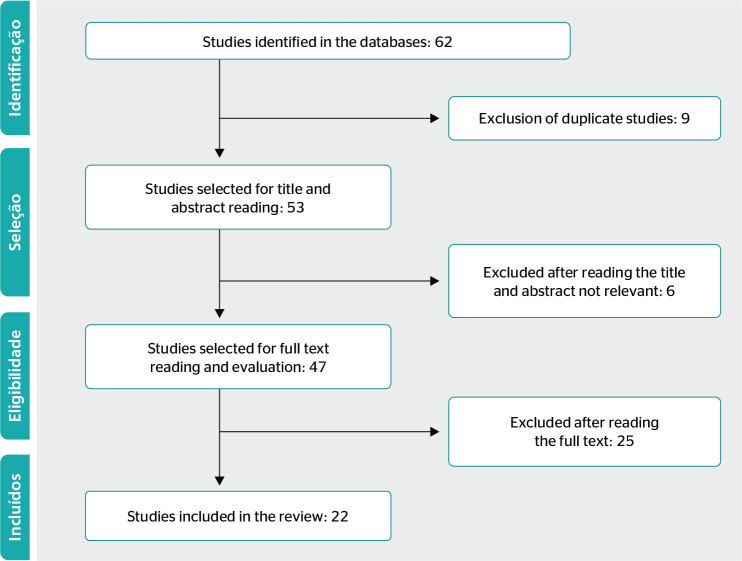
Primary study selection flowchart. PRISMA (Preferred Reporting Items for Systematic Reviews and Meta-Analyses). Maringá, PR, Brazil, 2020.

Publications found in the aforementioned databases were examined and duplicate articles were excluded. The titles and abstracts of all articles were read, and those that did not fulfill the eligibility criteria were excluded. After reading the full text of eligible articles, any that did not directly address the issue of retirement were excluded. The selection flowchart is shown in Figure 2.

The following data were retrieved from the 22 included articles: year of publication, author(s), language, objective, sample, data collection instrument (if any), type of data analysis, results, and conclusions. The studies were stratified by level of evidence according to Melnyk & Fineout-Overholt.^13^

## RESULTS AND DISCUSSION

A total of 22 studies were included in the review, which are described in Table 1 according to database, year of publication, title, author(s), country, and level of evidence.

**Table 1:** Summary of studies included in the review^*^

Id	Source	Year	Title	Author(s)	Country	LE
01	BVS	2018	“Prevalence and related factors of Active and Healthy Ageing in Europe according to two models: Results from the Survey of Health, Ageing and Retirement in Europe (SHARE)”	Bosch-Farré et al.^14^	Spain	IV
02	BVS	2018	“Health, education and employment status of Europeans aged 60 to 69 years: results from SHARE survey”	Augner^10^	Germany	IV
03	BVS	2018	“Predictors and estimation of risk for early exit from working life by poor health among middle and older aged workers in Korea”	Lee et al.^15^	South Korea	IV
04	BVS	2019	“Short-Term and Medium-Term Impact of Retirement on Sport Activity, Self-Reported Health, and Social Activity of Women and Men in Poland”	Biernat et al.^16^	Poland	IV
05	BVS	2019	“Quality of Life and Health: Influence of Preparation for Retirement Behaviors through the Serial Mediation of Losses and Gains”	Hurtado & Topa^17^	Spain	VI
06	BVS	2019	“Associations of childhood health and financial situation with quality of life after retirement - regional variation across Europe”	Börnhorst et al.^18^	Germany	IV
07	BVS	2019	“Evaluating the Effectiveness of the Health Management Program for the Elderly on Health-Related Quality of Life among Elderly People in China: Findings from the China Health and Retirement Longitudinal Study”	Hao et al.^19^	China	III
08	BVS	2017	“The interaction between individualism and wellbeing in predicting mortality: Survey of Health Ageing and Retirement in Europe”	Okely et al.^20^	UK	III
09	BVS	2017	“Papel de trabalho, carreira, satisfação de vida e ajuste na aposentadoria”	Boehs & Silva^21^	Brazil	IV
10	BVS	2017	“Common attributes in retired professional cricketers that may enhance or hinder quality of life after retirement: a qualitative study”	Filbay et al.^22^	UK	IV
11	BVS	2017	“Quality of Life of the Elderly Receiving Old Age Pension in Lesotho”	Mugomeri et al.^23^	Lesotho	IV
12	BVS	2017	“Retirement preparation program: evaluation of results”	Pazzim & Marin^24^	Brazil	III
13	BVS	2016	“Qualidade de vida na concepção de docentes de Enfermagem aposentadas por uma Universidade Pública”	Liberatti et al.^25^	Brazil	VI
14	BVS	2016	“Professional women ‘rebalancing’ in retirement: Time, relationships, and body”	Loe & Johnston^26^	USA	IV
15	BVS	2015	“Redirection: An Extension of Career During Retirement”	Cook^27^	Canada	VI
16	BVS	2016	“A Revised Australian Dietary Guideline Index and Its association with Key Sociodemographic Factors, Health Behaviors and Body Mass Index in Peri-Retirement Aged Adults”	Thorpe et al.^28^	Australia	IV
17	PubMed	2015	“Mid-life occupational grade and quality of life following retirement: a 16-year follow-up of the French GAZEL study”	Platts et al.^29^	UK	IV
18	PubMed	2019	“Associations between prevalente multimorbidity combinations and prospective disability and self-rated health among older adults in Europe”	Sheridan et al.^30^	USA	IV
19	PubMed	2016	“Self-reported change in quality of life with retirement and later cognitive decline: prospective data from the Nurses’ Health Study”	Vercambre et al.^31^	France	IV
20	PubMed	2019	“Do psychosocial factors modify the negative association between disability and life satisfaction in old age?”	Puvill et al.^32^	Denmark	IV
21	PubMed	2016	“Social group memberships in retirement are associated with reduced risk of premature death: evidence from a longitudinal cohort study”	Steffens et al.^33^	Australia	III
22	PubMed	2018	“Social isolation and multiple chronic diseases after age 50: A European macro-regional analysis”	Cantarero-Prieto et al.^34^	USA	IV

* Maringá/PR - 2021.

BVS = Biblioteca Virtual em Saúde; LE = level of evidence; PubMed = Biblioteca Nacional de Medicina dos Estados Unidos.

**Figure 3:**
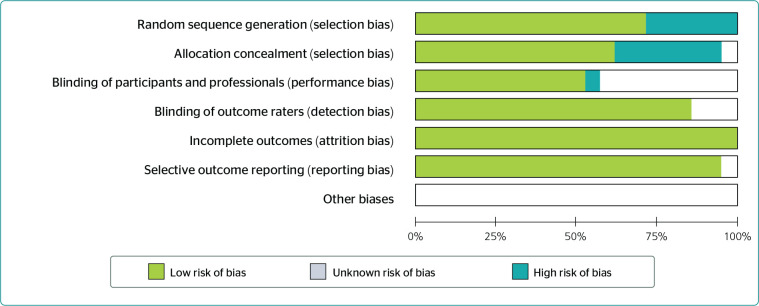
Risk of bias graph: analysis of the authors’ judgment on each risk of bias item, presented as percentages for all included studies. Source: Review Manager 5.4.1 - Maringá/2021.

The review was conducted according to the PRISMA (Preferred Reporting Items for Systematic reviews and Meta-Analysis) protocol, a 27-item checklist for improving the quality of systematic reviews and meta-analyses.^35^ According to Cochrane Collaboration recommendations, the studies underwent risk of bias assessment in seven domains: random sequence generation, allocation concealment, blinding of participants and professionals, blinding of outcome evaluators, incomplete outcomes, selective outcome reporting, and other sources of bias.^36^ Review Manager 5.4.1 was used to help summarize risk of bias judgment for clinical trials and create figures representing the results (Figure 3).

The included studies evaluated criteria such as physical and cognitive health, self-reported health, social support, and depressive symptoms, in addition to lifestyle habits such as diet, exercise, smoking, alcohol use, etc. Adjustment and adaptation to retirement depended on the health and quality of life of the retiree.^17^ The factors that influenced the quality of life of retirees were organized into the categories described below.

### FINANCIAL SITUATION

The fear of financial difficulties during retirement can interfere with healthy aging.^14^. A Brazilian study of women retired from a public university found that they feared their financial situation would change and that they would not be able to maintain their standard of living after retirement. The women reported that a good financial situation was closely associated with better quality of life.^25^ In Brazil, changes in the social security system have raised concerns about the financial future. Since retirees do not feel safe living on their retirement alone, they tend to look for ways to supplement their income. Another Brazilian study found that life satisfaction was characterized by its financial dimension, ie, having sufficient resources to “enjoy life” in retirement.^21^ In contrast, a study of professionals from public companies who deal with the general public found that they tend to retire early, even if they are unprepared for the psychological and financial transition, due to job dissatisfaction and poor quality of life.^36^

From another point of view, a study in Lesotho of retired rural residents whose main source of income was their pension found that the main determinants of low quality of life were related to their environment: the majority of respondents did not have a bathroom in their homes, had a house made of mud and straw, used firewood or paraffin as their main source of energy, and had limited access to information and financial resources.^23^ These factors were even more pronounced when compared by sex: women in Lesotho had lower education levels and held lower socioeconomic status than men.^23^

A study of 13,092 older Europeans found that the quality of life during retirement was influenced by the socioeconomic conditions they experienced in childhood, ie, those with better financial situations in childhood had greater access to quality education, which resulted in higher income in their careers and better quality of life in retirement.^18^

Thus, the results of articles included in this review show that retirement income is associated with quality of life. However, a German retrospective cohort study of 586 retirees found that retirement affected financial resources less than expected. The relationship between retirement and the economy could not be described as a single average trend, thus labor market differences remain despite retirement.^37^

### SOCIAL LIFE

Old age leads to considerable changes beyond biological decline.^14^ Some seniors experience retirement as “forgetfulness”,^21^ while others see it as a way to “enjoy life”, and others interpret it as the end of recognition, the rupture and loss of social life, a feeling of no longer belonging,^21^ a loss of purpose, of dwindling friendships, and fear of exclusion.^26^ In addition, increased social seclusion in retirement can restrict physical activity, leading to greater mobility difficulties, which can accelerate the aging process and increase the incidence of chronic diseases.^11^

A Spanish study of 244 retirees from different professional classes found that well-being in retirement was related to personal resources, such as knowledge, self-esteem, and social contacts. Retirees reported low self-esteem and decreased contact with friends and colleagues due to retirement.^17^ Accordingly, studies have found that younger older adults have higher rates of social isolation, which seems related to retirement and the stigma it represents, especially in industrial societies. Retirees feel devalued, unproductive, unengaged in social and work activities, and miss the social interaction provided by work, leading to a great emotional void.^38^

A Polish study on the impact of retirement found that, compared to those who were still working, men retired in the short term (2 years) had less zest for life, less social interaction, began consuming greater amounts of alcohol, and complained about health problems that made everyday life difficult. In the long term, the same population showed declines in physical and sexual activity and increased chest pain, fatigue unrelated to work, and health dissatisfaction^16^. Thus, it is important for health professionals to develop strategies, such as specific preventive interventions, in order to prepare pre-retirees for the retirement process.

According to a follow-up study of 11,293 French retirees, social position and job function also influence quality of life in retirement, ie, the more important the job, the greater the quality of life (physical and mental health, wealth, and social status) in retirement.^29^ Improving personal interactions, strengthening bonds, and engaging in intellectual activities are other factors that influence quality of life.^31^

### HEALTH CONDITIONS

Chronic diseases directly affect the active and healthy aging of retirees. According to a study of South African retirees, medication dependence due to chronic disease was a predictor of lower quality of life.^23^ A European study found that older adults who still worked felt less lonely and had fewer physical limitations and chronic diseases.^11^ Another European study found that 50% of retired older adults had at least 2 chronic diseases, which was closely related to depressive symptoms,^30^ although the depressive symptoms could also have been influenced by the type of jobs the workers performed.^11^

Some retirees interpret retirement as a period of “waiting to die”.^21^ Especially after the age of 75, older adults have lower healthy aging scores^14^, indicating that the greatest impediment to healthy aging is chronic disease or disability, followed by decreased social activity and cognition levels.^14^ A Korean study of retirees (mean age 55 years) found that early retirement was often motivated by health problems, low education, lower family income, and manual labor. These retirees had medium or low perceived health scores, in addition to risky health behaviors, such as smoking and alcohol use.^15^

In this context, an Australian study found that the reasons people retire were health problems or the need to take care of a friend or family member. These reasons were associated with worse mental health outcomes, which demonstrates the importance of considering these factors when developing strategies to keep older adults in the labor force or help them transition to retirement.^34^

In a study of retired cricket players in the United Kingdom, participants reported that, despite osteoarthritis and chronic pain due to their profession, they felt fortunate for their career and were satisfied with their quality of life after retirement.^22^ The players said that because they were aware of body changes they were able to adapt their choices and accept the limitations imposed by their career and advancing age.^22^

Women in the United States characterized retirement as a time to rebalance themselves, monitor their health, perform self-care, and become aware of their age.^26^ Several studies have linked positive self-perceived health to better quality of life.^18,28^

### RETIREMENT PREPARATION PROGRAMS

Retirement preparation programs have shown positive results regarding factors that influence retirement. In a study of workers in pre-retirement from a public institution in southern Brazil, the participants showed greater autonomy and freedom in decision-making, greater involvement in activities outside of work, and investment in personal projects and volunteering.^24^ However, women have shown greater acceptance for such programs, which can be explained by their greater investment in healthy behavior and interpersonal relationships.^24^

Given the above, retirement preparation programs should be developed to increase the satisfaction and success of retirees. A study of pre-retired Brazilian federal civil servants (aged 51 to 67 years) from 4 agencies obtained significant results with a retirement preparation program consisting of 13 group sessions. Among participants, the intervention led to an increase in activities such as talking about retirement plans with their spouse/partner, taking care of their health, and considering savings issues.^19^

U.S. nurses had higher quality of life scores when they replaced work with social activities and volunteering. Cognitive capacity decrease more slowly among nurses who reported higher quality of life, suggesting positive future effects.^31^

## CONCLUSIONS

Certain factors may be associated with quality of life in retirement. One is finances, since greater economic power means less fear and insecurity and better quality of life. Another is social life, since decreasing social activity often leads to psychosomatic problems, increased addiction, and feelings of abandonment. However, some retirees have reported greater freedom and social possibilities in retirement, which are protective factors against chronic and psycho-emotional disease. Other important factors include health, since preexisting chronic diseases at retirement can affect active aging, leading to disability, in addition to retirement preparation programs, which, despite low acceptance, effectively facilitate the retirement process, re-signifying free time in retirement, stimulating autonomy in decision-making, and presenting a range of new activities after leaving the work force.

The results of this review suggest that research should be conducted with retirees from different cultures and professional categories who have different levels of education and income to help identify and analyze other factors that could influence life satisfaction in retirement. These investigations should determine the association between well-being, self-rated health, and aging, focusing on physical function rather than the presence of diseases or disabilities. Monitoring workers during pre-retirement and in the years after retirement is also suggested, including comparison of individuals who participated in retirement programs and those who did not.
